# CHARACTERISING POLYPHARMACY IN THE VERY OLD: FINDINGS FROM THE NEWCASTLE 85+ STUDY

**DOI:** 10.1093/geroni/igz038.3262

**Published:** 2019-11-08

**Authors:** Laurie Davies, A Kingston, Adam Todd, Barbara Hanratty

**Affiliations:** 1 Newcastle University, Newcastle upon Tyne, United Kingdom; 2 School of Pharmacy, Newcastle University, England, United Kingdom

## Abstract

Polypharmacy is potentially harmful and under-researched amongst the fastest growing section of society, the very old (85+). This study aimed to characterise polypharmacy using data from the Newcastle 85+ Study – a prospective cohort of people living in north east England who turned 85 in 2006 (n=845). The prevalence of polypharmacy was examined using cut-points of 0, 1, 2-4, 5-9 and ≥10 medicines – so-called ‘no polypharmacy’, ‘monotherapy’, ‘minor polypharmacy’, ‘polypharmacy’ and ‘hyperpolypharmacy.’ Crosstabulations and Upset plots identified the most frequently prescribed medicines and medication combinations within these categories, at age 85. Mixed effects models assessed whether gender and socioeconomic position were associated with prescribing changes over time (age 85.5-90.5 years). Polypharmacy (49.6%) was more common than minor polypharmacy (24.6%) and hyperpolypharmacy (16.0%). Within these categories, cardiovascular, non-opioid analgesic and gastrointestinal medications were most frequently prescribed. Medication combinations were many and varied. Aspirin and statins were most commonly co-prescribed amongst people with minor polypharmacy and polypharmacy. Non-opioid analgesics, statins, aspirin and loop diuretics were most common in hyperpolypharmacy. Medications varied by gender and socioeconomic status. Nitrates and oral anticoagulants were more frequently prescribed for men. Bisphosphonates, analgesics and antidepressants were more common in women. Cardiovascular medications, including loop diuretics, were more frequently prescribed for socioeconomically disadvantaged people (25th centile Index of Multiple Deprivation (IMD)), with tricyclic antidepressants and selective beta-2 agonists more common amongst the least disadvantaged (>75th centile IMD). By highlighting prescribing patterns, this study informs our understanding of how polypharmacy may contribute to adverse outcomes in later life.

